# Modulation of cigarette smoke induced alterations by aqueous *Ocimum sanctum* leaf extract in pulmonary tissue of rodents

**DOI:** 10.1038/s41598-022-26152-8

**Published:** 2023-09-22

**Authors:** Pavitra Ranawat, Navdeep Kaur, Ashwani Koul

**Affiliations:** Department of Biophysics, Panjab University, Chandigarh, 160014 India

**Keywords:** Biological techniques, Cell biology, Drug discovery, Molecular biology

## Abstract

Smoking has been associated with an increased risk of asthma, lung cancer, cardiovascular diseases, chronic bronchitis, and a massive amount of oxidative stress. The present study was undertaken to determine the modulatory effects of Holi Basil/Tulsi, (*Ocimum sanctum)* leaf extract on cigarette smoke-induced pulmonary damage in mice. Cigarette smoke (CS) inhalation increased the levels of pulmonary lipid peroxidation, and reactive oxygen species and decreased the levels of glutathione. Histoarchitectural alterations and enhanced tissue lactate dehydrogenase (LDH) activity in pulmonary tissue was distinctly indicative of damage. Enhanced mucin production was also observed through mucicarmine and Alcian Blue-Periodic Acid Schiff (PAS) staining. Increased expression of MUC5AC was also observed. Alterations in the lung were also evident through FTIR studies. Administration of *Ocimum sanctum* leaf extract (80 mg/kg b.w) to CS exposed mice ameliorated these alterations to a greater extent. These findings are suggestive of the fact that *Ocimum sanctum* leaf extract effectively modulated CS-induced deleterious effects on pulmonary tissue.

## Introduction

Cigarette smoking causes cardiovascular diseases like atherosclerosis^1^, male infertility^2^, stroke^3^ cancer of the larynx, mouth, and oesophagus^4^ .The organ most vulnerable to smoking-induced damage, are the lungs^5^. Cigarette smoke contains highly reactive free radicals^6^ heavy metals As, Cd, K, Sb, and Zn^7^ and various PAHs^8^. The tar phase of cigarette smoke contains free radicals like the quinone/hydroquinone (Q/QH2) complex which reduces oxygen to produce superoxide eventually leading to hydrogen peroxide and hydroxyl radicals. The gas phase of cigarette smoke contains small oxygen- and carbon-centered radicals that are much more reactive than are the tar-phase radicals. The major underlying problem in cigarette smoking is the oxidative stress caused due to high amount of reactive oxygen species. Oxidative stress plays an important role in airways diseases such as chronic obstructive pulmonary disease (COPD)^9^. Mainstream and side-stream smoke each have about 1X10^(16)^ radicals per cigarette which are remarkably long-lived. A steady-state concentration of these radicals is produced by the slow oxidation of nitric oxide to the more reactive nitrogen dioxide, followed by the reaction of nitrogen dioxide with reactive organic molecules in smoke^10^. To counter their effect, the body is endowed with antioxidants. Any shift in the critical oxidant/antioxidant balance could result in an increase in the peroxidative stress and may lead to cellular damage^11^.

Thus, the use of antioxidants is the most rational way to counter the damage induced by cigarette smoke. *Ocimum sanctum* also known as Holy Basil or Tulsi belongs to the plant family Lamiaceae. It has made an important contribution to the field of science due to its large number of medicinal properties. *Ocimum sanctum* is reported to have anticancer activity by many^12,13^. Its radioprotective role has also been reported^14,15^. It is shown to exert antifungal properties by disrupting ergosterol biosynthesis^16^. Linoleic acid in *Ocimum sanctum* shows antibacterial activity against staphylococcus^17^. Major component of *Ocimum sanctum* is said to be eugenol. Other than this carvacrol, sesquiterpene, hydrocarbon caryophyllene, cirsilineol, circimaritin, isothymusin, apigenin, androsameric, linoleic acid, orientin, vicenin, Ursolic acid, apigenin, luteolin, apigenin-7-O-glucuronide, luteolin-7-oglucuronide are also identified from *Ocimum sanctum*^18^. Various flavonoids and phenols present in the *Ocimum sanctum* contribute to its antioxidant properties^19^.

Thus, the antioxidant activity of the *Ocimum sanctum* makes it a potent defense against oxidative stress in the body. Keeping this in mind, the present study was designed to determine the ameliorative potential of *Ocimum sanctum* leaf extract against cigarette smoke-induced alterations in pulmonary tissue of mice.

## Materials and methods

### Animals and treatment

Male laca mice (25–35 g) were taken from the Central Animal House, Panjab University, Chandigarh, and kept in polypropylene cages bedded with rice husk. They were given free access to clean drinking water and a standard animal pellet diet throughout the experiment. Proper ventilation and ambient room temperature of 25 ± 2 °C were maintained where the animals were kept for the experiment. The experimental protocols were approved by the Institutional Ethics Committee (IAEC), Panjab University, Chandigarh and conducted according to Indian National Science Academy Guidelines. The study is also reported in accordance with ARRIVE Guidelines. The animals were randomly divided into four groups (7–8 animals each) on the basis of the treatment they received.

**Figure 1:**
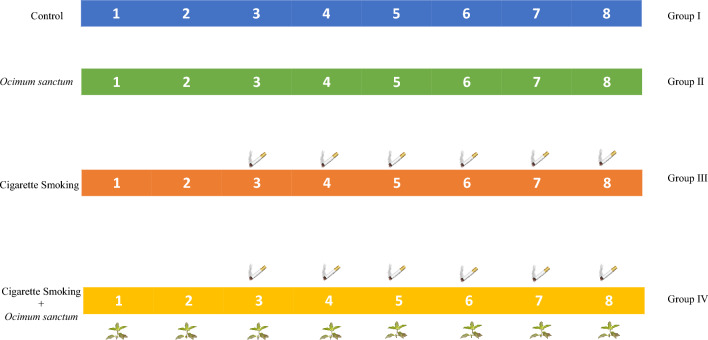
Treatment regimen.

Group I served as the **Control** and was exposed to fresh air. Group II (**OSE**) was administered with *Ocimum sanctum* leaf extract orally at a dose of 80 mg/kg body weight daily for a period of eight weeks. Group III was exposed to cigarette smoke (**CS**) inhalation as described by Koul et al.^20^ for six weeks. Group IV (**OSE + CS**) was exposed to cigarette smoke inhalation for six weeks and was co-administered with *Ocimum sanctum* leaf extract orally at a dose of 80 mg/kg for eight weeks. *Ocimum sanctum* leaf extract administration was started two weeks prior to cigarette smoke inhalation (Fig. 1).

### Passive cigarette smoke inhalation

Five to seven animals were exposed to CS at a time from commercially available cigarettes in an inhalation apparatus. The inhalation apparatus included a suction pump attached to a Perspex chamber (8.21 L). The chamber consisted of two inlets, one for smoke and another for fresh air respectively. To control the airflow a valve was attached to the outlet of the chamber. Animals were exposed to CS for 1 h daily. Each cigarette took approximately 6 to 8 min to burn completely. After exposure to smoke from one cigarette, a gap of 5 min was given. This procedure had been standardized in such a way that animals inhaled CS without any respiratory stress as is evident from carboxy-hemoglobin (CO-Hb) levels^20^.

### Preparation of aqueous leaf extract of *Ocimum sanctum*

The leaves of *O. sanctum* were dried in shade and finely powdered. The leaf powder (100 g) was then refluxed with 750 ml of double-distilled water for 1 h at 75–80  C. It was then cooled and filtered. This was repeated in three trials. The extracts were pooled and evaporated using a lyophilizer^21^.

Standard ABTS and DPPH assays were carried out with the extract to check for radical scavenging activity (Supplementary file 1).

### Reduced glutathione (GSH)

GSH was estimated according to the method described by^22^ which is based on the reduction of 5, 5-dithiobis-(2-nitrobenzoic acid) (DTNB) by the SH group of GSH to form one mole of 2-nitro-5-mercaptobenzoic acid per mole of –SH. The 2-nitro-5-mercaptobenzoic acid formed has a deep yellow color, which can be measured at 412 nm. The assay was performed within an hour of sacrificing the animal to avoid errors due to oxidation of GSH. The results were expressed as micromole of GSH/g tissue.

### Lactate dehydrogenase (LDH)

LDH activity was measured by the method of^23^. LDH catalyzes the reduction of pyruvate with NADH to form NAD^+^. A decrease in absorbance at 340 nm which is proportional to the LDH activity in the sample gives the rate of oxidation of NADH to NAD^+^. One unit of enzyme activity was defined as nanomoles of NADH consumed/min/mg protein using an extinction coefficient of 6.22 mM^−1^ cm^−1^.

### Lipid peroxidation (LPO)

Lipid peroxidation was estimated by method of^24^. The levels of MDA produced gives the intensity of oxidative stress. Cycloperoxides formed due to the deterioration of lipids form malondialdehyde (MDA). MDA forms a pink colored complex with thiobarbituric acid (MDA- TBA chromophore), which can be read at 532 nm.

### Reactive oxygen species (ROS)

Reactive oxygen species were determined using the method of^25^. ROS accumulation was detected with the carboxy-H_2_-DCFDA (Molecular Probes) staining method. This assay is based on the principle that the nonpolar, non-ionic dichlorofluorescein diacetate dehydrate (H_2_-DCFDA) crosses cell membranes and is enzymatically hydrolyzed into nonfluorescent dicholorofluorescein dehydrate(H_2_-DCFDA) by intracellular esterases. In the presence ROS, H_2_-DCF is rapidly oxidised to become highly fluorescent dichlorofluorescein (DCF) which is then measured.

### FTIR sample preparation

Freshly excised lung tissue was flash freezed in liquid nitrogen. The tissues were then lyophilized and the powdered sample was spread over KBr plate (IR inactive) and then made into pellets of 1.2 cm diameter using hand hydraulic press (Perkin Elmer, Germany). The FTIR spectra were obtained in the absorbance mode with FTIR spectrometer (Perkin Elmer, Germany). The records of 64 interferograms at a spectral resolution of 2 cm^−1^ and a sampling interval of 1 cm^−1^ were averaged for each spectrum. The whole data was then corrected with a background energy reading from a blank KBr pellet. Each spectrum was baselined using a polynomial function and then normalized to adjust the optical characteristics of each sample.

### Histopathological analysis and mucin histochemistry

Histopathological analysis of lung tissue was done using Hematoxylin and Eosin staining as described by^26^. Lungs were removed and immediately transferred to neutral formalin and allowed to fix for 12 h. Next, the tissue was dehydrated gradually in ascending series of ethanol. For embedding, the dehydrated samples were placed in benzene, then sequentially in 1:1 benzene: paraffin wax with two changes in pure wax before finally embedding. 5 µm thick sections were obtained using a manual hand-driven microtome and transferred to glass slides. These were then dewaxed in xylene, rehydrated in descending series of ethanol, and stained with Hematoxylin and Eosin. Stained sections were mounted in DPX and viewed under a light microscope. The tissue sections were also stained with Meyer’s Mucicarmine stain and Alcian Blue-Periodic Acid Schiff’s Stain to evaluate mucins and differentiate between acidic and neutral mucins.

### RNA expression studies

mRNA expression analysis by RT-PCR was performed using the INVITROGEN RT-PCR kit.

### Total RNA Isolation

Total RNA was isolated from mouse lungs using TRI-REAGENT. 50 mg tissue from different treatment groups was homogenized in 0.5 ml TRI-REAGENT using a hand homogenizer. The samples were kept at room temperature for 5 min after which 80 μl chloroform was added. This was mixed vigorously for about 15 s and the homogenates were then kept at room temperature for 10 min followed by centrifugation at 12,000 rpm for 15 min at 4 °C. Following centrifugation, the upper colorless aqueous phase containing RNA was collected. In order to precipitate RNA, an equal volume of isopropanol was added and after mixing, the samples were kept at room temperature for 10 min and spun thereafter at 12,000 rpm for 10 min at 4 °C. RNA precipitate so obtained was washed by adding 75% ice-cold ethanol and spinning at 7500 g for 5 min at 4 °C. After removing ethanol, the RNA pellet was briefly air-dried (not completely) and then dissolved in DEPC treated water. Purity, integrity, and concentration of the isolated RNA were checked by taking absorbance at 260 and 280 nm and finding their ratio. The concentration of RNA was estimated by using A_260_ = 1 = 40 μg/ml.

#### RT-PCR procedure

RT-PCR was done using specific primers for the respective genes. RT-PCR for β-actin was also done along with to rule out the experimental errors. INVITROGEN one-step RT-PCR kit was used for the purpose. 2 μg of total RNA was used in RT-PCR reaction from different groups. To this, the following reagents were added as follows: 10 μl 5 × Invitrogen one-step RT-PCR buffer, 2 μl dNTP mixture, 5 μl each of forward and reverse primers. (10 μM stock), 2 μl enzyme mix, and 1 μl RNase inhibitor (1 U/μl). Finally, PCR grade RNase-free water was added to make the total volume 50 μl. The components were mixed with a gentle vortex and centrifuged to collect all the components at the bottom of the tube. The PCR reaction was performed in the thermal cycler (Techne Ltd, England) using the following conditions: RT reaction was performed at 50 °C for 50 min and activation at 94 °C for 15 min. PCR was followed by 35 cycles of 94 °C (denaturation) for 45 s, Tm (annealing) for 45 s, 68 °C (extension) for 1 min. Finally, the products were incubated at 68 °C for 5 min to extend any incomplete single strands.

#### Agarose gel electrophoresis of PCR products

Final PCR products formed were analyzed on 1.5% agarose gel electrophoresis and densitometric analysis of the bands was done by Image J software (NIH, USA). The mean of four independent densitometric analyses of PCR product bands were determined for comparison of each analysis.

### Primer used

**Table:** 

CYP1A1		Tm(°C)
Forward primer	AGACCTCTACAGCTTCACACTTAT	59.3
Reverse primer	CTCAAATGTCCTGTAGTGCTCTTT	59.3

**Table:** 

MUC5AC		Tm(°C)
Forward primer	CTCCTGTGACATTATCCCATAAGC	61.0
Reverse primer	GAAAGTGTAGTAGGTGCCATCAAA	59.3

**Table:** 

β-actin		Tm(°C)
Forward primer	ATCCGTAAAGACCTCTATGC	55.3
Reverse primer	AACGCAGCTCAGTAACAGTC	57.3

### Statistical analysis

The data from individual groups were compared using Statistical Package for Social Sciences software (SPSS version 14.0) and the groups were presented as the mean ± S.D. Differences between groups were analyzed using a one-way analysis of variance (ANOVA) and a post hoc test. Significant Difference (LSD) test and minimum criterion for statistical significance was set at *p* < 0.05 for all comparisons.

## Results

**Figure 2:**
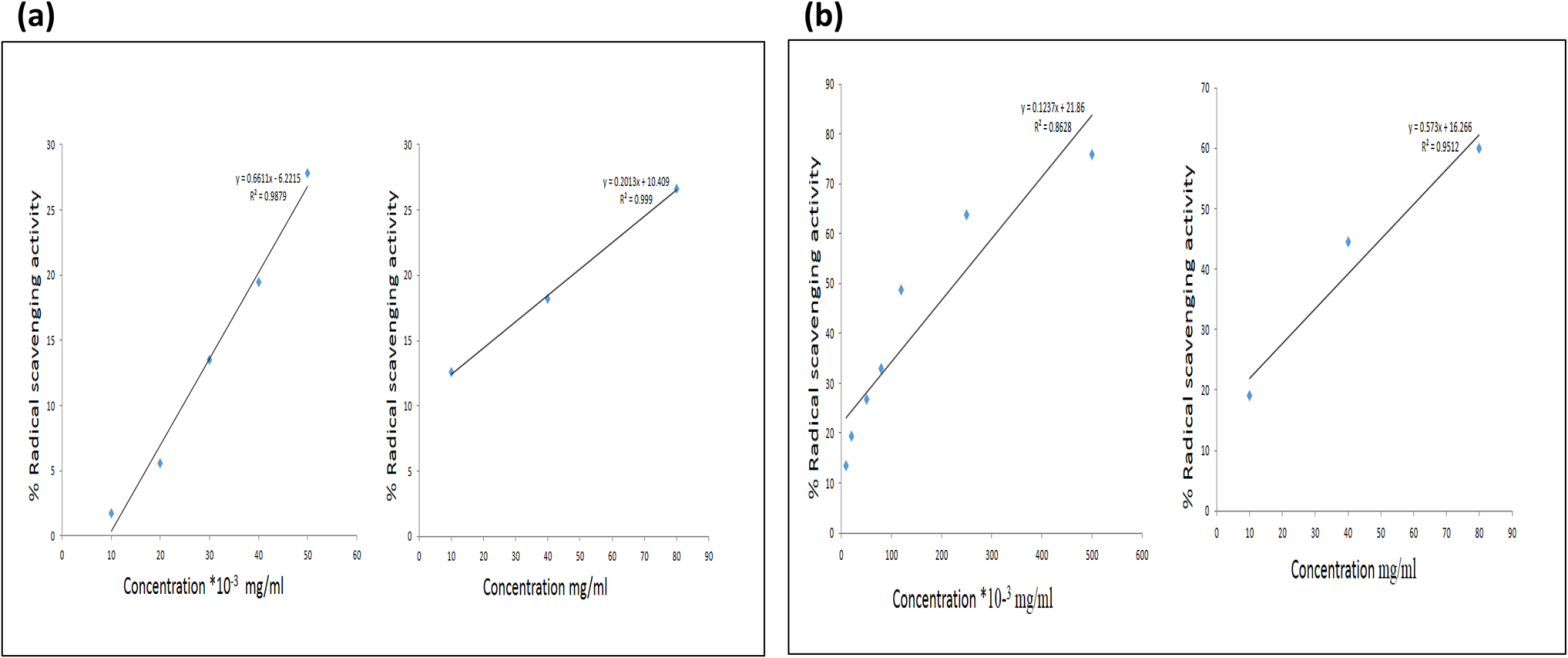
Graph depicting the total antioxidant capacity of ascorbic acid (reference compund) and *Ocimum sanctum* using (**a**) DPPH and (**b**) ABTS assay.

The radical scavenging activity of *Ocimum sanctum* leaf extract was evaluated by DPPH and ABTS assay (Fig. 2). A concentration dependent increase was observed in the activity of both ascorbic acid as well as aqueous extract of *Ocimum sanctum* against free radicals in DPPH assay along with ABTS assay respectively.

### Reduced glutathione level (GSH)

**Figure 3:**
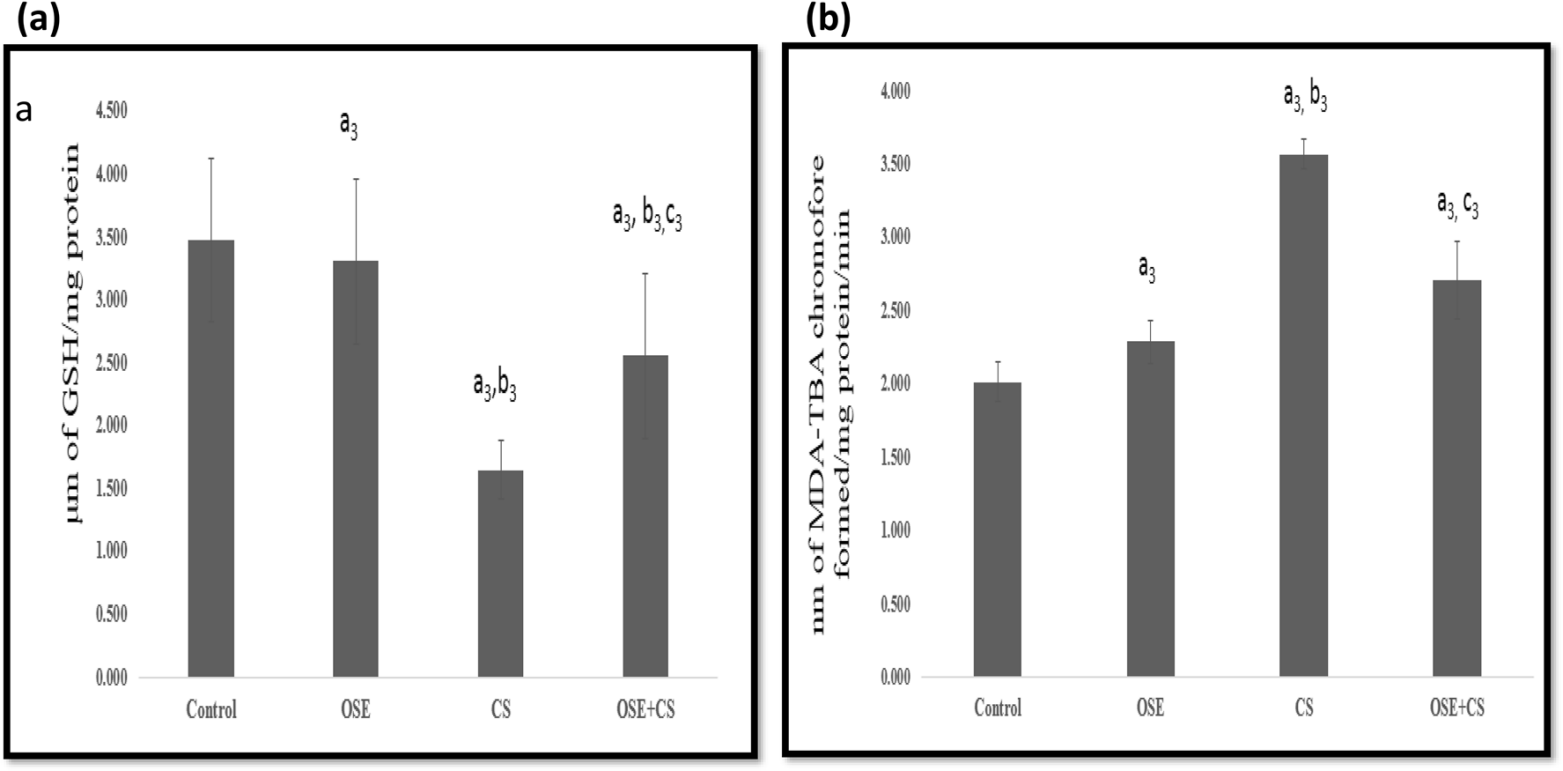
Effect of *Ocimum sanctum*, Cigarette smoke and co-treatment of Cigarette Smoke and *Ocimum sanctum* on (**a**) pulmonary reduced glutathione levels and (**b**) pulmonary MDA levels. Data is expressed as Mean±SD (n=5). Data is analyzed using one-way ANOVA followed by post hoc test. a_3_* p* ≤ 0.001 significant with respect to control group; b_3_* p* ≤ 0.001 significant with respect to Ocimum sanctum group (OSE); c_3_* p* ≤ 0.001 significant with respect to cigarette smoke group (CS). Units - μm of GSH/mg protein.

A significant decrease was observed in the GSH levels of pulmonary tissue from cigarette smoke-exposed group when compared to the control (*p* ≤ 0.001) and *Ocimum sanctum* treated group (*p* ≤ 0.001). Administration of *Ocimum sanctum* to cigarette smoke inhaling mice caused a significant increase in GSh levels when compared to the cigarette smoke-exposed group (*p* ≤ 0.001) Fig. 3a.

### Lipid peroxidation (LPO)

Cigarette smoke exposure caused a significant increase in pulmonary LPO levels when compared to the control (*p* ≤ 0.001) and *Ocimum sanctum* treated group (*p* ≤ 0.001). Administration of *Ocimum sanctum* to cigarette smoke inhaling mice caused a noteworthy decrease when compared to the cigarette smoke-exposed group (*p* ≤ 0.001) Fig. 3b.

### ROS

**Figure 4:**
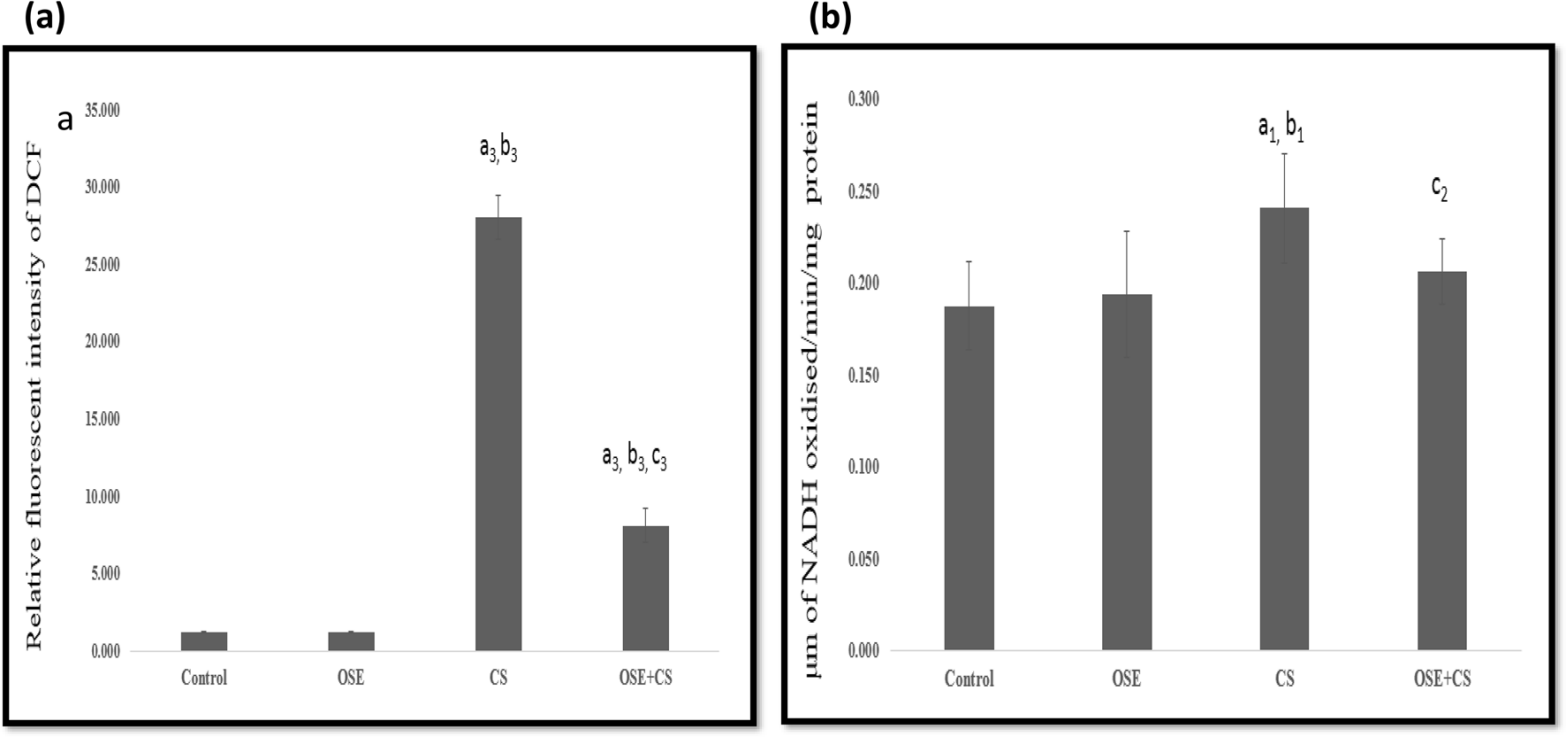
Effect of *Ocimum sanctum*, Cigarette smoke and co-treatment of Cigarette Smoke and *Ocimum sanctum* on (**a**) pulmonary ROS generation and (**b**) pulmonary Lactate Dehydrogenase activity. Data is expressed as Mean±SD (n=5). Data is analyzed using one-way ANOVA followed by post hoc test. a_1_* p* ≤ 0.05 significant with respect to control group; b_1_* p* ≤ 0.05 significant with respect to Ocimum sanctum group (OSE); c_2_* p* ≤ 0.001 significant with respect to cigarette smoke group (CS). Units - μm of NADH oxidised/min/mg protein.

A noteworthy increase was observed in ROS levels from cigarette smoke-exposed animals in pulmonary tissue (*p* ≤ 0.001) when compared to the control and *Ocimum sanctum* treated animals. *Ocimum sanctum* treatment to cigarette smoke inhaling mice caused a very significant decrease in ROS levels as compared to the cigarette smoke-exposed animals (*p* ≤ 0.001) Fig. 4a.

### Lactate dehydrogenase (LDH)

A considerable increase in LDH activity was observed in pulmonary tissue from cigarette smoke-exposed animals when compared to control (*p* ≤ 0.05) and *Ocimum sanctum* treated groups (*p* ≤ 0.05). Administration of *Ocimum sanctum* to cigarette smoke inhaling mice caused a noteworthy decrease in LDH activity when compared to the cigarette smoke-exposed group (*p* ≤ 0.01) (Fig. 4b).

### Histoarchitectural analysis

**Figure 5:**
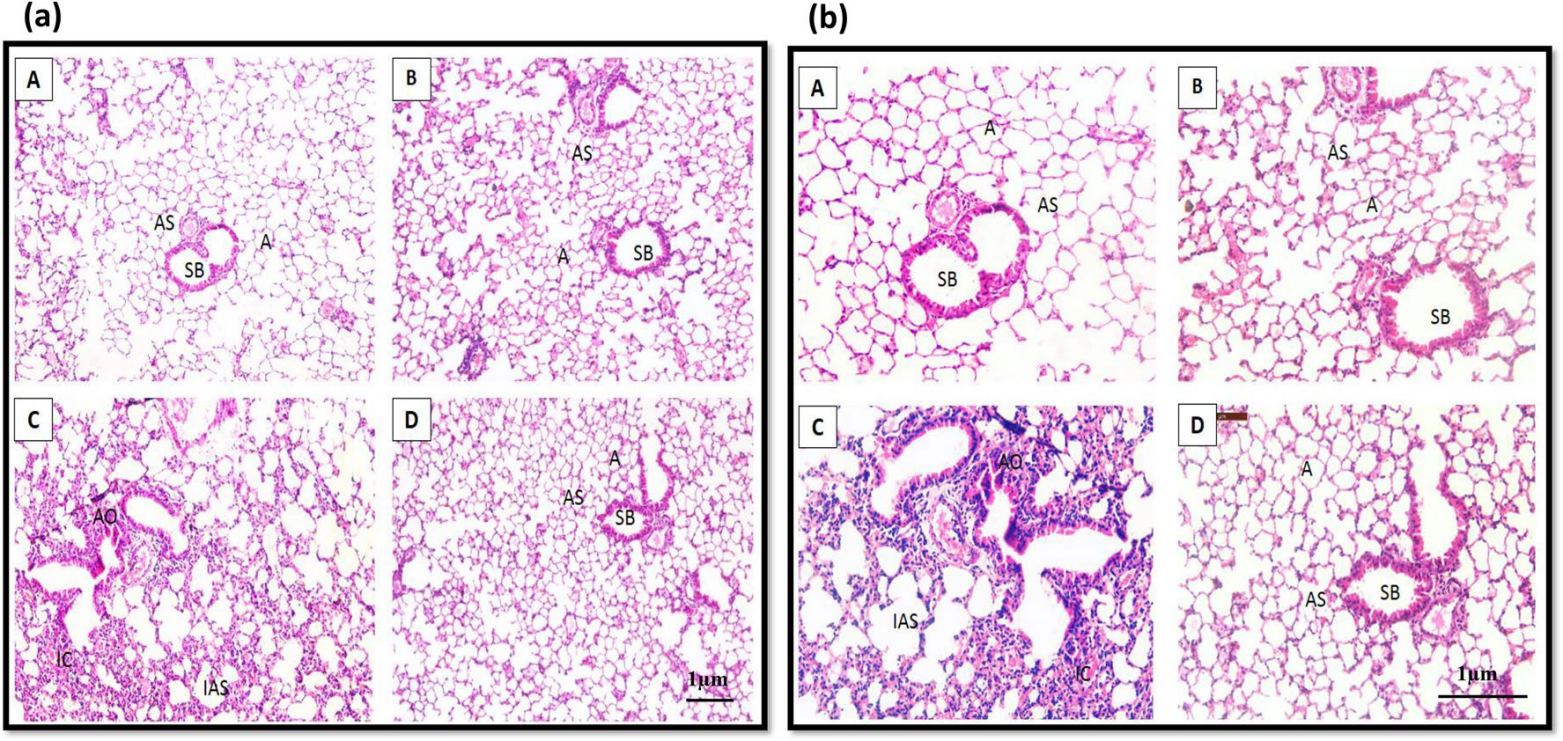
Hematoxylin and Eosin stained section of lung from animal of Control (A), *Ocimum sanctum* treated group (B) , Cigarette smoke exposed mice (C), *Ocimum sanctum* + cigarette smoke treated group (D) at (**a**) 100X and (**b**) 200X, showing small bronchiole (SB), alveolar sac (AS), alveolus (A), alveolar occlusion (AO), inflammatory cells (IC), increased alveolar space (IAS).

Lung tissue was analyzed for histoarchitectural changes in response to treatment with cigarette smoke, *Ocimum sanctum,* and co-treatment with *Ocimum sanctum* and cigarette smoke, (Fig. 5). The lungs of control mice showed normal histoarchitectural features. Cigarette smoke exposure caused alveolar destruction leading to increased alveolar space. Alveolar occlusion and thickening of alveolar walls were also observed. Interstitial the tissue inflammation was also visible.

Animals exposed to co-treatment of *Ocimum sanctum* and cigarette smoke did not show any deleterious changes in the pulmonary histoarchitecture. Multiple regions with normal histoarchitectural features were observed.

### Mucin histochemistry

**Figure 6:**
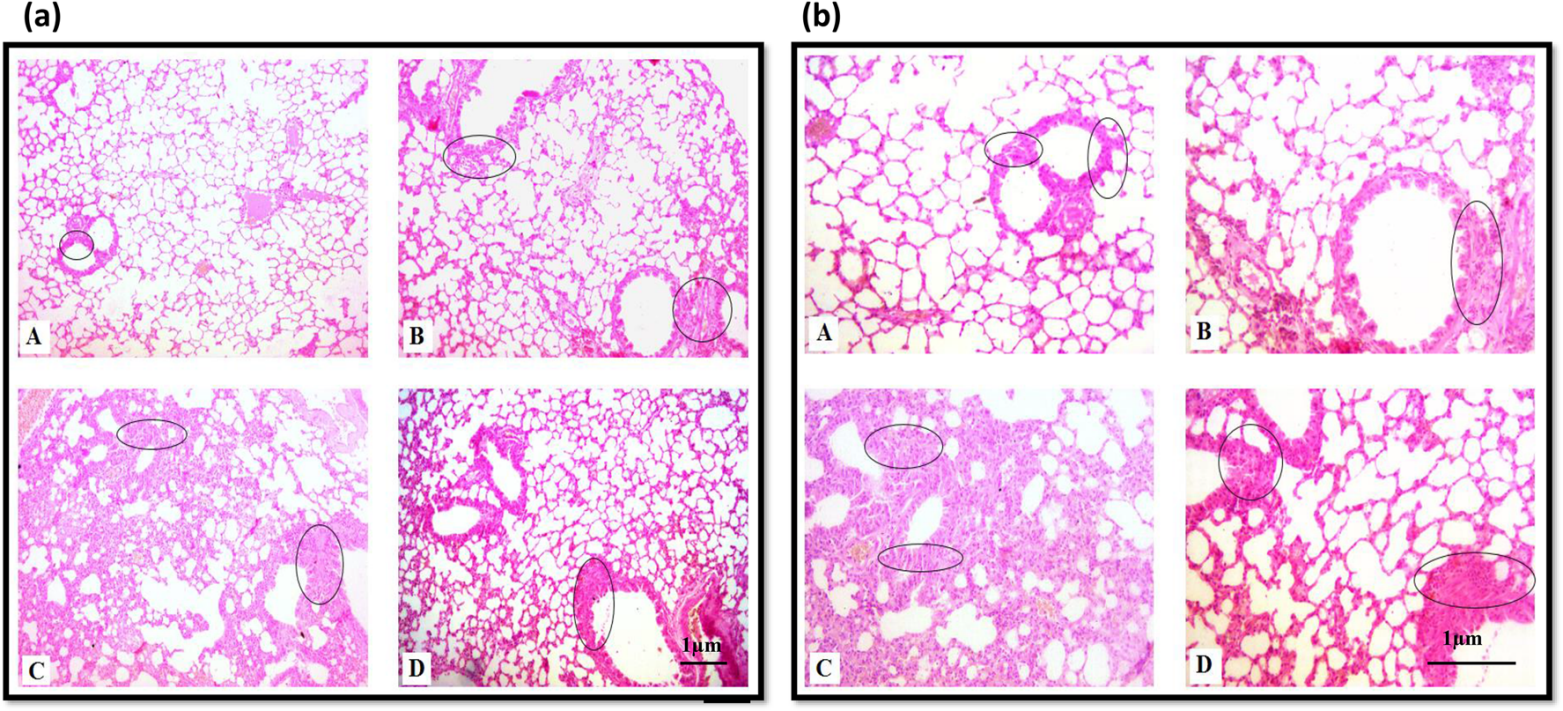
Mucicarmine stained section of lungs from control (A), *Ocimum sanctum* treated group (B), cigarette smoke exposed group (C), *Ocimum sanctum* + cigarette smoke exposed group (D) at (**a**) 100X and (**b**) 200X.

**Figure 7:**
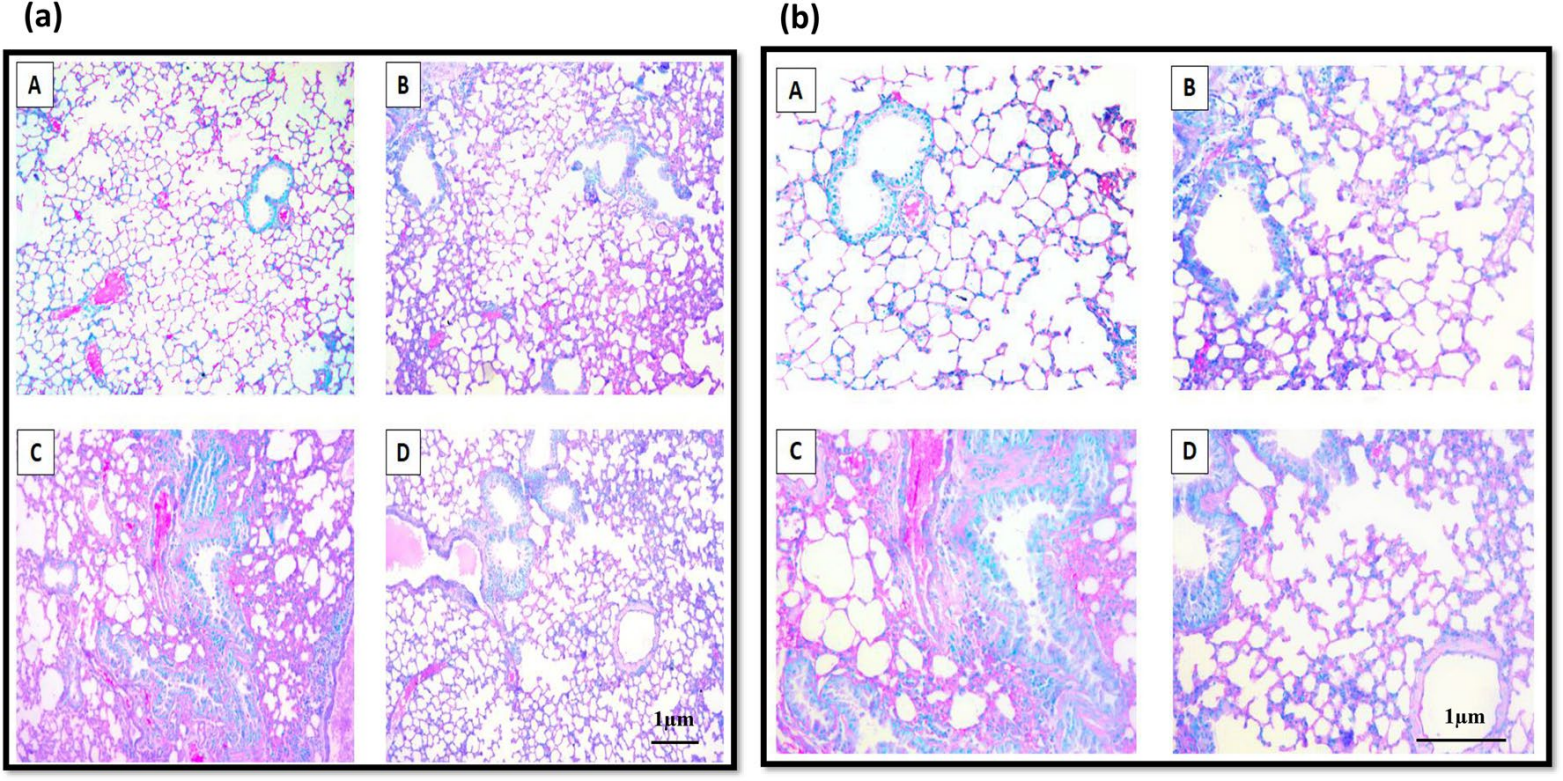
AB-PAS stained section of lung from Control animal (A), *Ocimum sanctum* treated group (B), cigarette smoke exposed group (C), *Ocimum sanctum* + cigarette smoke exposed group (D) at (**a**) 100X and (**b**) 200X; neutral mucins are stained by PAS (pink) and acidic mucins are stained by Alcian blue (blue).

Lung tissue sections were stained with Meyer’s mucicarmine (Fig. 6) and Alcian blue-Periodic Acid Schiff (AB-PAS) (Fig. 7) to demonstrate mucin production. Some mild mucicarmine-stained alveolar and bronchiolar regions were observed in lung tissue from the control group. Cigarette smoke exposure enhanced mucin production as indicated by increased mucicarmine staining in alveolar and bronchiolar regions. Areas in the lung of animals exposed to cigarette smoke revealed enhanced production of neutral mucins (intense Schiff’s stain). Increased mucin production was revealed in bronchiolar regions. *Ocimum sanctum* and cigarette smoke co-treatment group also revealed increased mucin production. However, it was less apparent than in the cigarette smoke alone group.

### mRNA expression of CYP1A1

**Figure 8:**
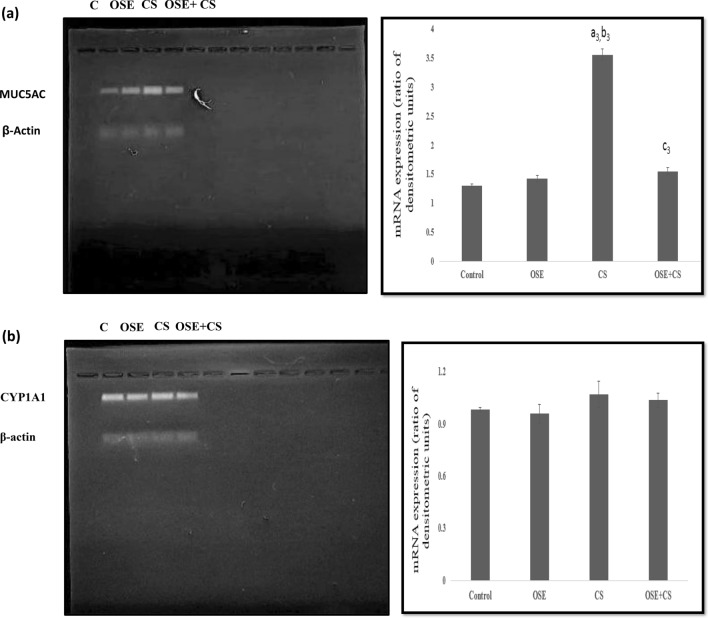
(**a**) mRNA expression of the MUC5AC in lungs. (**b**) mRNA expression of the CYP1A1 in lungs. Data is expressed as Mean±SD (n=5). Data is analyzed using one-way ANOVA followed by post hoc test. a_3_* p* ≤ 0.001 significant with respect to control group; b_3_* p* ≤ 0.001 significant with respect to Ocimum sanctum group (OSE); c_3_* p* ≤ 0.001 significant with respect to cigarette smoke group (CS). Units – mRNA expression (ratio of densitometric units).

The expression of the CYP1A1 gene in the lungs was observed. The expression was observed to be increased in cigarette smoke inhaling mice when compared to the control and *Ocimum sanctum* treated mice. *Ocimum sanctum* treatment to cigarette smoke inhaling mice was observed to decrease the expression of CYP1A1 as compared to cigarette smoke inhaling mice Fig. 8a.

### mRNA expression of MUC5AC

There was a marked increase in expression of MUC5AC in lungs in cigarette smoke-exposed animals as compared to control. A decrease was observed in expression in the OSE + CS group when compared to the cigarette smoke inhaling group. The expression in control and *Ocimum sanctum* treated mice was almost similar to each other (Fig. 8b).

### Analysis of the FTIR spectra

**Figure 9:**
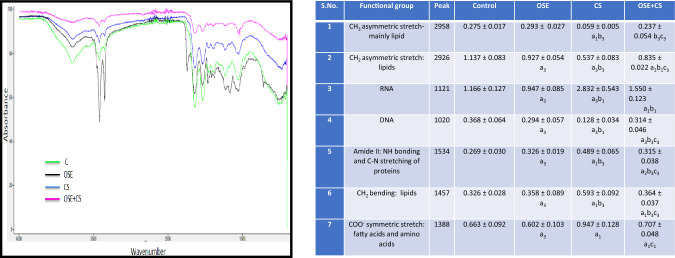
FTIR spectrum of pulmonary tissue of Control, *Ocimum sanctum*, Cigarette smoke and co-treatment of Cigarette Smoke and *Ocimum sanctum* (Y axis—Absorbance, X axis—Wave number cm^−1^). Data is expressed as Mean±SD (n=5). Data is analyzed using one-way ANOVA followed by post hoc test. a_3_* p* ≤ 0.001, a_2_* p* ≤ 0.01, a_1_* p* ≤ 0.05 significant with respect to control group; b_3_* p* ≤ 0.001, b^1^* p* ≤ 0.05 significant with respect to Ocimum sanctum group (OSE); c_3_* p* ≤ 0.001, c_1_* p* ≤ 0.05 significant with respect to cigarette smoke group (CS).

FTIR spectroscopy is a vibrational spectroscopic technique that can be used to optically probe the molecular changes associated with diseased tissue. The method is employed to find more conservative ways of analysis to measure characteristics within tissue and cells that would allow accurate and precise assignment of the functional groups, bonding types, and molecular conformations^27^. Alterations were observed in area under peaks at 1121 cm^−1^, 1020 cm^−1^, 2926 cm^−1^, 2958 cm^−1^ and under spectral region 1350 cm^−1^–1550 cm^−1^ Fig. 9.

### Statistical analysis

The data from individual groups was compared using Statistical Package for Social Sciences software (SPSS version 14.0) and the groups were presented as the mean ± S.D. Differences between groups were analyzed using one way analysis of variance (ANOVA) and tukeys post hoc test. Minimum criterion for statistical significance was set at *p* < 0.05 for all comparisons.

## Discussion

Glutathione (GSH), a ubiquitous tripeptide thiol, is a vital intra- and extracellular protective antioxidant against oxidative/nitrosative stresses, which plays a key role in the control of proinflammatory processes^28^. A decrease in the levels of GSH in pulmonary tissue was observed by^29,30^. Administration of nicotine which is a component of cigarette smoke showed a decrease in liver GSH^31^. In the present study also a significant decrease was observed in GSH levels due to CS exposure. This decrease can be attributed to the conjugation of glutathione to the xenobiotics (free radicals, highly electrophilic radicals) in an attempt to eliminate those from the body, consequently decreasing the GSH levels in the body. However, administration of *Ocimum sanctum* leaf extract to CS exposed mice increased the GSH levels as compared to the CS group. Application of *Ocimum* leaf extract on skin papilloma showed a twofold elevation of reduced glutathione content in the skin of mice^32^. Improved GSH levels were also observed in isoproterenol-administered rats fed with *Ocimum sanctum* as compared to rats treated with isoproterenol alone^33^.

Lipid peroxidation is a marker of oxidative damage. An increase in lipid peroxidation levels was seen in the CS group as compared to the control group. The increase in LPO is attributable to the free radicals generated due to cigarette smoke which is also observed by the increased ROS in the CS group in the present study. Increase in malondialdehyde (MDA levels) which is a product of lipid peroxidation was also reported by^34,34^ also established an increase in lipid peroxidation due to cigarette smoke. Thiol-reactive stable compounds in CS were stated to activate NADPH oxidase and increase endothelial free radical production, which could lead to excessive ROS generation and enhanced lipid peroxidation. *Ocimum sanctum* treatment to the CS groups decreased the lipid peroxidation levels as compared to those in CS groups. A decrease in LPO due to administration of aqueous leaf extract of *Ocimum sanctum* to rats with high-fat diet-induced injury is reported^36^. This decrease in LPO by *Ocimum sanctum* administration could be attributed to the presence of oleanic acid and urosolic acid in *Ocimum sanctum*^37^.

Lactate dehydrogenasethatn enzyme converts lactate to pyruvate and vice versa as it converts NAD + to NADH and back^38^. An increase in LDH levels was observed in CS groups as compared to the control group in the present study. An increase in LDH activity is a marker of tissue injury which is also evident from the histopathological observations. An increase in LDH activity due to cigarette smoke has also been reported by^39^. *Ocimum sanctum* is reported to decrease the LDH levels in diabetic rats^40^ and rats with doxorubicin-induced toxicity^41^. A decrease in LDH activity due to *Ocimum sanctum* administration to CS exposed mice was also observed presently.

*Ocimum sanctum* administered mice showed normal histoarchitectural features like small bronchiole, alveolar sacs, alveolus, and alveolar walls similar to control. Cigarette smoke-exposed mice showed a high degree of alveolar damage. There was increased alveolar spaces due to alveolar destruction. Also, thickening of the alveolar walls was observed. Inflammatory cells were also visible indicating increased tissue inflammation. Similar changes due to cigarette smoke were also reported by Koul and co-workers in 2015. Alveolar destruction is also observed in COPD^42^ and emphysema^43^. The destruction could be due to the presence of high ROS generation causing oxidative stress and tissue injury. An increase in LDH activity also indicated tissue injury. *Ocimum sanctum* treatment to cigarette smoke-exposed mice, however, revealed little alveolar destruction. There were also normal regions as compared to the cigarette smoke-exposed mice. This could be due to recovery by virtue of the antioxidant properties of *Ocimum sanctum* treatment as also indicated by ROS level estimations^37^.

Benzopyrene, most abundant poly aromatic hydrocarbon present in cigarette smoke forms DNA adducts leading to carcinogenesis in the longer run. The transformation of benzopyrene to the adduct formation is triggered by the increased expression of CYP1A1^44^. This is why the expression of CYP1A1 was studied. There was a marked increase in its expression in case of cigarette smoke-exposed group as compared to the control animals as also observed by McLemore and co-workers in their study^45^. A decrease in expression was observed in OSE + CS group as cthe ompared tosmoke-exposedoke exposed group which could be attributable to the antioxidants present in *Ocimum sanctum* leaf extract*.*

Mucin overproduction is one of the first defense mechanisms of the lungs against cigarette smoke. It is regulated by MUC5AC gene. Lung sections from control animals showed mild acidic and neutral mucin production. There was enhanced mucin production in cigarette smoke exposed mice. Also, the MUC5AC gene expression was observed to be upregulated in cigarette smoke exposed animals as compared to control and OSE group presently. Mucin overproduction due to cigarette smoke is also reported by various others^46^. Increased mucin production is attributed to the attempt of mucocilliary clearance of foreign particles after cigarette smoke exposure. Lungs from *Ocimum sanctum* + cigarette smoke treated mice showed enhanced mucin production but it was apparently less when compared to the cigarette smoke exposed group. Also, expression of MUC5AC was less in CS exposed mice treated with OSE which shows that *Ocimum sanctum* is helpful in restoring pulmonary histoarchitechture.

The 1250–1000 cm region in the FTIR spectra is sensitive to nucleic acid backbone conformation due to vibrations along the sugar–phosphate chain^47^. Significant alterations were observed in area under this region in peaks 1121 cm^−1^ and 1020 cm^−1^ in case of CS exposed animals. This is suggestive of DNA damage in the tissue due to CS. DNA damage due to cigarette or tobacco smoking has been reported by many^48–50^. In accordance to this study many have also reported that benzopyrene is notorious in forming DNA adducts through CYP1A1 gene^51,52^. The damage could be due to the high ROS generation. Bending modes of –CH_2_ and –CH_3_ groups as well as C–N stretching of fatty acids and proteins were depicted by spectral region of 1550–1350 cm^−1^. Alterations were observed at the peaks 1388 cm^−1^ 1451 cm^−1^ and 1534 cm^−1^ in ciggrate-exposed animals. Alterations in proteome profile due to cigarette smoke have been reported. Mainly changes in proteins involved in inflammation, apoptosis and xenobiotic metabolism were reported^53^. Remarkable alterations were also observed at the peaks centered at 2958 cm^−1^ and 2926 cm^−1^ which depicts asymmetric stretching modes of –CH_2_ and –CH_3_ respectively in the cigarette smoke exposed animals and *Ocimum sanctum* treated group as compared with control group. This is indicative of lipid peroxidation in the tissue damaging the plasma membrane. Decrease in CH_2_ and CH_3_ stretching and lipid peroxidation due to cigarette smoke was also reported.

Taking into consideration the above-mentioned findings, the present study therefore concludes that *Ocimum sanctum* leaf extract has huge potential in ameliorating cigarette smoke induced deleterious alterations in the pulmonary tissue. However, further studies on identifying the underlying mechanism involved in conferring protection needs to be taken up. Also, the screening and therapeutic evaluation of bioactive compounds from Ocimum sanctum leaf extract need to be taken up further. This will lead to the development of a therapeutic moiety for the treatment of cigarette smoke induced alterations in various tissues including Lungs.

### Supplementary Information

Supplementary Information.

## Data Availability

All data generated or analysed during this study are included in this published article.
