# Supplementary material for: Modulation of cigarette smoke induced alterations by aqueous Ocimum sanctum leaf extract in pulmonary tissue of rodents
Source: Sci Rep. 2023 Sep 22;13:15806. doi: 10.1038/s41598-022-26152-8 (PMC10517011; doi:10.1038/s41598-022-26152-8)
Supplement: Supplementary file 1 — Supplementary Information. [file 41598_2022_26152_MOESM1_ESM.docx]

|  | Control | OSE | CS | OSE+CS |
| --- | --- | --- | --- | --- |
| GSH  (μm of GSH/mg protein) | 3.474 ± 0.645 | 3.304 ± 0.659  a_3_ | 1.646 ± 0.229  a_3,_ b_3_ | 2.553 ± 0.623  a_3,_ b_3,_c_3_ |
| LPO  (nm of MDA-TBA chromofphore formed/mg protein/min) | 2.014 ± 0.132 | 2.286 ± 0.147  a_3_ | 3.506 ± 0.102  a_3,_ b_3_ | 2.707 ± 0.261  a_3,_ c_3_ |
| ROS  (Relative fluorescent intensity of DCF) | 1.185 ± 0.021 | 1.227 ± 0.031 | 28.083 ± 1.401  a_3,_ b_3_ | 8.088 ± 1.104  a_3,_ b_3,_ c_3_ |
| LDH  (μm of NADH oxidised/min/mg protein) | 0.188 ± 0.024 | 0.194 ± 0.034 | 0.241 ± 0.030  a_1,_ b_1_ | 0.206 ± 0.018  c_2_ |

**Raw data pertaining to the levels of GSH, LPO, ROS and LDH**

Data is expressed as Mean±SD (n=5). Data is analyzed using one-way ANOVA followed by post hoc test. ^a^_3_p≤0.001, ^a^_1_p≤0.05 significant with respect to control group; ^b^_3_p ≤0.001, ^b^_1_p≤0.05 significant with respect to *Ocimum sanctum* group; ^c^_3_p≤0.001, ^c^_2_p≤0.01 significant with respect to cigarette smoke group.
